# ﻿Systematic reinstatement of the Sumatra endemic species *Mangiferasumatrana* Miq. (Anacardiaceae)

**DOI:** 10.3897/phytokeys.199.80727

**Published:** 2022-06-09

**Authors:** Fitmawati Fitmawati, Erwina Juliantari, Mega Silvia

**Affiliations:** 1 Department of Biology, Faculty of Mathematics and Natural Sciences, Universitas Riau, Pekanbaru, Riau, 28293, Indonesia Universitas Riau Riau Indonesia; 2 Plant Biology Graduate Program, Department of Biology, Faculty of Mathematics and Natural Sciences IPB University, Jl. Raya Dramaga, Bogor, West Java, 16680, Indonesia Faculty of Mathematics and Natural Sciences IPB University Bogor Indonesia

**Keywords:** Accepted name, barcoding DNA, morphology, palynology, phylogeny

## Abstract

*Mangiferasumatrana* Miq. is an endemic species from Sumatra. The taxonomic status of *M.sumatrana* remains unclear and is currently treated as a synonym of *M.laurina*. The present study employed morphological and palynological characters and molecular analyses to address the delimitation between the two species. Pollen observations were carried out with a Scanning Electron Microscope (SEM). Phylogenetic relationships were investigated using the ITS and the *trn*l-F intergenic spacer markers. *M.sumatrana* differs from *M.laurina* by having pyramidal panicles with a low density of pale yellow flowers pale, sepals 3–3.5 × 1.7–2 mm, fruit roundish to flattened with pale yellow pulp, a rough fibre texture, and pollen with a prolate spheroidal shape. The MP phylogenetic tree showed a divergent boundary between the two species suggesting that *M.sumatrana* could be an independent species, not a variety of *M.laurina*. The present study supports the view that these two taxa can be treated as different species.

## ﻿Introduction

Mango (*Mangifera* spp.) has become a major fruit crop of the tropics and subtropics, particularly in Asia. The mango has always been one of the most important fruit crops and it has been considered the ‘king of fruits’. The genus *Mangifera* is one of the 73 genera belonging to the Anacardiaceae family in the order Sapindales. The latest classification of *Mangifera* by Kostermans and Bompard (1993) describes 69 species.

Morphologically, closely-related *Mangifera* are quite difficult to distinguish, leading to species complexity and misidentification. This is due to the continuity of the characters and the high morphological plasticity of *Mangifera*, as well as the diversity of species boundaries (Fitmawati and Hartana 2010). Continuity of characters or plasticity due to interspecific hybridisation is common in *Mangifera* and can occur due to a chromosomal match between *Mangifera* species, i.e. species are allotetraploids with 40 chromosomes, and these have polembrionic seeds (Litz 2004).

According to Kostermans and Bompard (1993), based on morphological characteristics, there is a species boundary between *M.laurina* Bl., *M.indica* L. and *M.lalijiwa* Kosterm., so that these three *Mangifera* species are separated, but for *M.sumatrana* Miq., which is identical to *M.laurina* Bl. and, until now, the taxonomic status of *M.sumatrana* is synonymous (POWO 2019). Before then, this species was treated as a synonym of *M.longipes* Griff. by Mukherjee (1953) and Hou (1978).

According to Konchummen (1996), *M.laurina* is a variation of *M.indica* and is a synonym of *M.indica.* Phenetic analysis, based on morphological characters, also showed that *M.sumatrana* Miq. has a close relationship with *M.indica* L. and *M.laurina* Bl (Fitmawati et al. 2013). However, based on molecular analysis using the ITS sequence by Fitmawati et al. (2016), the results identified that *M.sumatrana* Miq. did not form a clade with either *M.indica* L. or *M.laurina* Bl. *M.sumatrana* is rarely found in nature, so it is important to clarify its taxono­mic status.

Differences of opinion regarding the taxonomic status of *M.sumatrana* and its relatives are caused by differences in the sources of taxonomic evidence used as the basis for compiling different classifications and the rationale for classifying these plants is also different. Therefore, a more comprehensive source of evidence is needed to strengthen the taxonomic status of *M.sumatrana* and its relatives.

Conflicts have also developed amongst taxonomists regarding the taxonomic status of the species *M.sumatrana* due to the great rarity of the species. Therefore, we propose that this species needs to be evaluated using morphological methods, palynological characteristics and molecular phylogeny to confirm the identity of *M.sumatrana*. We hope this study can provide an outstanding example for re-evaluating synonyms for *M.sumatrana*.

## ﻿Materials and methods

Fresh leaf samples of *M.sumatrana* were collected from Sumatra, Indonesia. We have been doing exploration since 2013–2017. The voucher specimens of *M.sumatrana* (sheets: HR20130073, HR20130094, HR20160096, HR20170124) were generated and deposited at the Herbarium Riauense, Indonesia.

### ﻿Morphological and palynological analysis

The morphological characters observed in this study were qualitative and quantitative characters comprising stems, leaves, fruits and seeds. Morphological comparisons were made through herbarium studies and field observations. Herbarium studies were conducted in Herbarium Riauense, ANDA, BO and Kew (https://powo.science.kew.org/). A total of 2000 *Mangifera* spp. and 30 *M.sumatrana* collections number loaned from the following herbaria were examined for morphological data. Morphological characters are referred to as descriptors of mango (International Plant Genetic Resources Institute 2006) and Kosterman and Bompard 1993). We evaluated the conservation status of species using IUCN Red List categories (IUCN 2012). Pollen observations were carried out with a Scanning Electron Microscope (SEM) and consisted of preparation, mounting, coating, photographing of pollen and data analysis. Pollen grains were prepared in glycerine jelly and measured using an eyepiece (ocular) with a scale and then the measurement results were converted into micrometre units.

### ﻿Molecular methods and phylogenetic relationship analyses

Samples used in this study represent each section of *Mangifera* species obtained from Genbank. Fresh leaves of *M.sumatrana* used in this study were collected from Sumatra. Two genera from Anacardiaceae were used as an outgroup (Table 1). The DNA was amplified in a specific target area using the internal transcribed spacer (ITS) and the *trn*L-F intergenic spacer (IGS) marker. DNA extraction using the CTAB method of Doyle and Doyle (1987) with modification, was undertaken by using ethanol 96% for about 24 h at 4 °C. Barcoding sequence amplification was done through the PCR technique. The genomic DNA was amplified using universal primers ITS5F (5’-GGAAGTAAAAGTCGTAACAAGG-3’) and ITS4R (5’-TCCTCCGCTTATTGATATGC-3’) (White et al. 1990) (for the entire ITS regions, nrDNA) and primer E (5’-GGTTCAAGTCCCTCTATCCC-3’) and primer F (5’-ATTTGAACTGGTGACACGAG-3’) (Small et al. 2004) (for the entire *trn*L-F intergenic spacer region, cpDNA). PCR products were sent to First Base Laboratories, Malaysia. PCR Clean-Up was then used to purify the amplified products by Gel Extraction, depending on visualisation results for Single Pass DNA Sequencing.

**Table 1.** Sources of Mangifera sequences and their locality.

Species	Genbank Acc. No.	Locality	Species	Genbank Acc. No.	Locality
ITS sequences			*trnL-F* sequences		
*M.sumatrana* Miq.	MF990366	Indonesia	*M.sumatrana* Miq.	MF997590	Indonesia
*M.indica* L.	KX347960	Indonesia	*M.indica* L.	KY392616	Indonesia
*M.zeylanica* (Bl) Hook. f.	KX347962	Indonesia	*M.zeylanica* (Bl) Hook. f.	MF997591	Indonesia
*M.laurina* Bl.	MF678498	Indonesia	*M.laurina* Bl.	KY392609	Indonesia
*M.lalijiwa* Kosterm.	MF678504	Indonesia	*M.lalijiwa* Kosterm.	MF997587	Indonesia
*M.torquenda* Kosterm.	MF990365	Indonesia	*M.quadrifida* Jack.	KY392614	Indonesia
*M.quadrifida* Jack.	MF678511	Indonesia	*M.foetida* Lour.	MF997585	Indonesia
*M.casturi* Kosterm.	MF678493	Indonesia	*M.odorata* Griff.	MF945595	Indonesia
*M.foetida* Lour.	MF678506	Indonesia	*M.kemanga* Bl.	MF919594	Indonesia
*M.odorata* Griff.	KX347957	Indonesia	*M.andamanica* King	AB598013	India
*M.kemanga* Bl.	MF990368	Indonesia	*M.camptosperma*	AB598010	India
*M.oblongifolia* Hook. f.	AB071682	Thailand	*M.flava* Evrard.	MF945595	India
*M.gedebe* Miq.	AB071681	Thailand	*M.griffithi* Hook. f.	AB598012	Vietnam
*M.macrocarpa* Bl.	AB071688	Thailand	*M.reba* Pierre	KY067415	Vietnam
*M.sylvatica* Roxb.	AB071689	Thailand			
*M.cochinchinensis* Engler.	AB071675	Thailand			
*M.griffithii* Hook. f.	AB071685	Thailand			
*M.flava* Evrard.	AB071679	Thailand			
*M.pentandra* Hooker f.	AB071684	Thailand			
*M.pajang* Kosterm.	MF444896	India			

DNA sequences were aligned with ClustalW Multiple Alignment used Molecular Evolutionary Genetics Analysis (MEGA) software (Tamura et al. 2013; Kumar et al. 2016). Phylogenetic relationships analysis was performed using the Maximum Parsimony (MP) with the PAUP programme (Swofford 2002).

## ﻿Results and discussion

### ﻿Morphology

#### ﻿ Mangifera sumatrana

Taxon classificationPlantaeSapindalesAnacardiaceae

Miq. first published in Fl. Ned. Ind. 1(2): 630 (1859)

CCB9EDCA-218D-5115-B96E-3599AD9C7437

Fig. 1

##### Type.

Indonesia. Sumatra, Riau, Pekanbaru, tropical lowland, alt. 32 m, 3 October 2016, *Fitmawati 152* (holotype HR20130073!).

##### Diagnosis.

**Figure 1.**
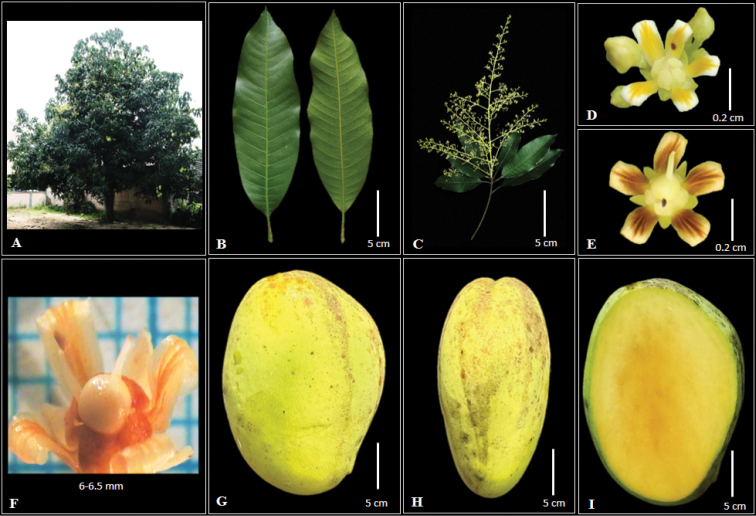
*Mangiferasumatrana***A** habit **B** adaxial and abaxial surface of leaf **C** pyramidal panicles **D** flowers **E** flowers after anthesis; **F** ovary with swollen disc **G** roundish fruit **H** flattened fruit **I** pulp.

**Figure 2.**
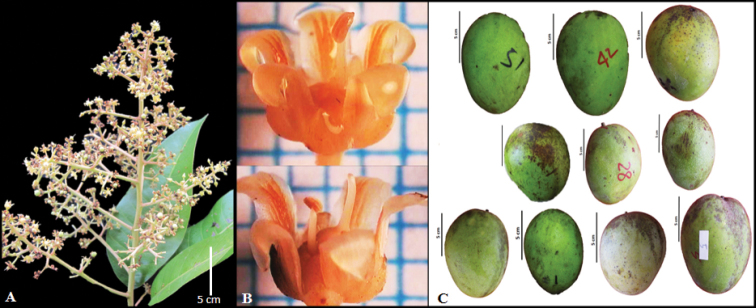
*Mangiferalaurina***A** conical panicles **B** flowers **C** roundish thick fruit.

*Mangiferasumatrana* has been considered as a synonym of *Mangiferalaurina* Bl. The distinctive characteristics of the *M.sumatrana* are panicles pyramidal and not dense, large and flat fruit, prominent fruit beak type, a quantity of fibre in pulp and high stone. *M.laurina* panicles are conical and dense, with small and thick fruit, round in shape and fruit break type is perceptible (Figs 1, 2).

##### Description.

Tree up to 40 m tall and 100–140 cm in diam., growth habit spreading, bark brownish-white with cream sap, the shoot brownish-yellow and crown semi-circular. ***Leaves*** dark green, scattered, semi-drooping on branch, chartaceous, oblong-ovate, apex acuminate, base acute, both surfaces smooth, 14.9–15.4 × 4.51–5 cm, thickness 0.12–0.2 cm, mid-rib 13.7–14.2 cm in length, above and below mid-rib prominent, nerves 21–23 pairs, areola reticulation dense, slightly prominent, two branches. Petiole 2.8–3 cm in length, 0.19–0.22 cm in diameter. ***Panicles*** terminal, semi-erect, yellowish-cream, pyramidal, 9.5–12 cm long, 14.30–15.55 g, non-glomerulate, low flower density. ***Flowers*** pale yellow with light yellow tinge, 5-merous, after anthesis, pale yellow with orangish-yellow tinge, 0.1–0.2 g, 6–6.5 × 5.5–6.2 cm. Bract yellowish-green, 5, 2.6–3.1 × 1.4–1.6 mm, broadly triangular acuminate, even and hairy, both dorsal and ventral smooth. Sepals light green, 5, 3–3.5 × 1.7–2 mm, broad ovate, acute and hairy and smooth. Petals pale yellow, 5, 5–5.4 × 2–2.3 mm, curved-reflexed outwards, elliptic, apex blunt, not hairy, ridges 5. Disc swollen, broader than ovary. Stamen fertile 1, 2.5–2.8 mm long, staminodes 4–5, filaments adnate to the base, 0.7–0.78 × 0.4–0.5 mm. Ovary rather round, lateral-frontal. Stylus slightly to the side and curved, 2–2.5 mm long. ***Fruits*** pale yellow, roundish flattened, thickness 0.2–0.3 cm, apex round, 160.41–182 g, 10.8–11.6 × 4.51–5.4 cm, 5.44–6 cm, skin surface texture smooth, non-waxy, density of lenticels on fruit skin sparse, beak pointed, sinus shallow, slope of fruit central shoulder rising and then rounded, fruit stalk insertion oblique, neck prominence absent. Pulp yellow, texture soft, adherence intermediate, quantity of fibre low, 6.13–6.4 cm long, juicy and sweet. 15.5° Brix. Stones oblong, 23.51–25 g, 8.7–9 × 4.22–4.5 cm, 1.14–1.3 cm thickness, fibre texture rough, adherence of fibre to stone weak, veins on stone depressed and pattern of stone venation forked. Polyembryony, 2.22–3 g. ***Leaf anatomy*** Anomocytic stomata type. Simple epidermis. Simple palisade mesophyll. Upper mid-rib of *M.sumatrana* has convex and lower mid-rib has concave shape.

##### Distribution and habitat.

*M.sumatrana* is an endemic species only found in lowland areas in Sumatra (less than 100 m a.s.l.), collected in southern Sumatra and central Sumatra, but is more commonly found in Riau Province, Sumatra, Indonesia.

##### Chemotaxonomy.

In addition, several compounds from the alkaloid, alkane, amino acids, benzene, benzoic acid and fatty acyl groups are only found in *M.sumatrana* Miq. Conversely, several compounds from the phenolic group (gallic acid), amino acids, benzene and benzoic acid are only found in *M.laurina* Bl (Fitmawati et al. 2021). Therefore, it can be reported that *M.sumatrana* Miq. is not a synonym of *M.laurina* Bl and contradicts the morphological classification of Kostermans and Bompard (1993).

##### Notes.

Geographically, the distribution of *M.sumatrana* and *M.laurina* is also different. *M.sumatrana* is found in lowland areas of Sumatra (less than 100 m a.s.l.), while *M.laurina* is a cosmopolitan species and is not only found in Sumatra, but also in the Maleisiana area, especially in the highlands (altitude up to 2000 m a.s.l.) (Fitmawati et al. 2013).

### ﻿Palynology

Mukherjee (1953) investigated the pollen morphology of mango and 12 other *Mangifera* species. Their pollen grains were tricolpate of almost the same size. Mondal (1982), cited in Kostermans and Bompard (1993), attempted to correlate pollen morphology with taxonomic relationships of 17 *Mangifera* species, based upon different characteristics of the exine and sporoderm. They demonstrated that all of the species of section II (subgenus Limus) possess a coarse exine, whereas there was no clear correlation with pollen type in species within section I (subgenus Mangifera).

**Figure 3.**
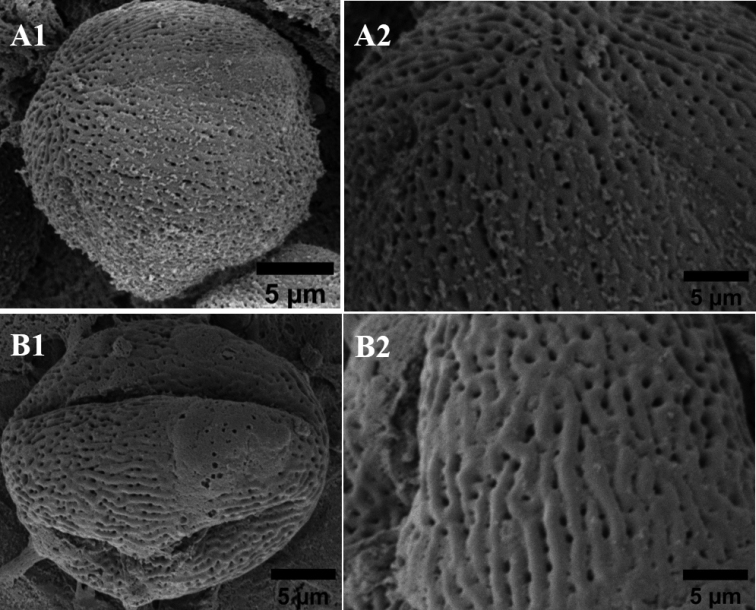
Pollen polarity and pollen aperture of the four types *Mangifera*, by electron microscope (A1–B1). Surface ornamentation of *Mangifera* pollen (A2–B2) **A***M.sumatrana* and **B***M.laurina*.

*Mangifera* are closely related morphologically and are quite difficult to distinguish, causing differences of opinion amongst experts regarding the taxonomic position of several *Mangifera* species. Therefore, more comprehensive and stable additional data are needed to strengthen the taxonomic status of *Mangifera*, namely using micromorphological pollen characters.

Based on the results of the study, there were five similarities in the characteristics of pollen morphology, namely pollen monad unit, angular polar view, circular/oval equatorial view, isopolar pollen polarity and tricolpate pollen aperture type, while the differences were pollen size, pollen shape and pollen ornamentation, polar diameter, equatorial length and exine thickness.

The relationship, based on a study of pollen micromorphology, shows that the pollen characteristics of *M.sumatrana* are very different from *M.indica*, while the difference between *M.sumatrana*, *M.laurina*, and *M.odorata* lies in the type of pollen ornamentation. *M.laurina* has the closest relationship with *M.odorata*. The results of this study can be a source of supporting evidence in clarifying the taxonomic status of *M.sumatrana* and showing that it differs from its relatives.

*M.sumatrana* has a striate-microreticulate ornamentation type, while *M.laurina* has a striate-reticulate ornamentation type, so that this pollen ornamentation feature can be a source of new taxonomic evidence for refuting the theory of Kostermans and Bompard (1993) which states that *M.sumatrana* is a synonym of *M.laurina*, based on morphological characteristics. This statement is also supported by research conducted by Fitmawati et al. (2018) which states that *M.laurina* and *M.sumatrana* are different and *M.laurina* is not a synonym for *M.sumatrana*, based on an analysis using ITS. This finding can be a source of supporting evidence in clarifying the taxonomic status of *M.sumatrana* and showing that it differs from its relatives (Table 2).

**Table 2.** Morphological and palynological differences between *Mangiferasumatrana* and *M.laurina*.

**Taxonomic traits**	***Mangiferasumatrana***	***Mangiferalaurina***
Panicle shape	Pyramidal	Conical
Panicle density	Low (14.30–15.55 g)	Medium (15.56–16.81 g)
Flowers’ colour	Pale yellow	Yellow-orange
Bractea	Yellowish-green (2.6–3.1 × 1.4–1.6 mm)	Green (2–2.5 mm × 1.1–1.3 mm)
Sepal size	3–3.5 × 1.7–2 mm	1.3–1.8 mm × 0.7–1 mm
Fruit shape	Roundish flattened	Roundish thicked
Fruit stalk insertion	Oblique	Vertical
Fruit neck prominence	Absent	Slightly prominent
Pulp colour	Pale yellow	Yellow-orange
Fibre texture in the pulp	Rough	Soft
Pollen ornamentation type	Striate-microreticulate	Striate-reticulate

### ﻿Phylogenetic relationship analysis

ITS sequences were obtained for all 24 species of *Mangifera* and two genera from Anacardiaceae were used as an outgroup. Alignment samples yielded 672 nucleotide sites distributed in the ITS region. The aligned ITS contained 452 (67.2%) conserved sites, 123 (18.3%) variable informative sites and 97 (14.5%) parsimony-informative site characters that were assumed to be informative for phylogenetic analysis using the parsimony method. The research resulted in a length of 369 steps and had a consistency index (CI) and retention index (RI) of 0.726 and 0.690, respectively (Table 3).

**Table 3.** Properties of the two candidate DNA barcoding loci in *M.sumatrana* with its relative species.

Parameter	ITS regions	trnL-F IGS	ITS+ trnL-F IGS
Sequences length	672	411	1582
Conserved sites (%)	67.20	90.75	89.60
Variable informative sites (%)	18.30	5.35	5.69
Parsimony-informative sites (%)	14.50	3.50	4.62
Tree length	534	60	221
Consistency index (CI)	0.72	0.67	0.91
Retention index (RI)	0.69	0.50	0.80

*trn*L-F IGS sequences were obtained for all 14 species of *Mangifera* and two genera from Anacardiaceae were used as an outgroup. Alignment samples yielded 411 nucleotide sites distributed in the *trn*L-F IGS. The aligned ITS contained 373 (90.75%) conserved sites, 22 (5.35%) variable informative sites and 16 (3.5%) parsimony-informative characters that were assumed to be informative for phylogenetic analysis using the parsimony method. The analysis resulted in a length of 369 steps and had a consistency index (CI) and retention index (RI) of 0.67 and 0.50, respectively.

**Figure 4.**
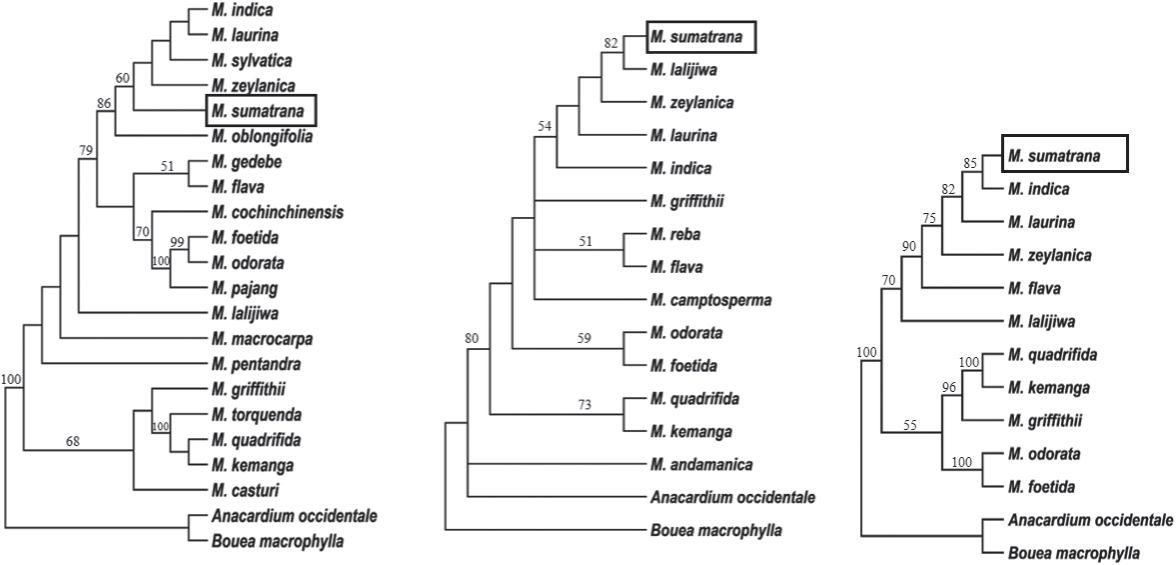
Phylogenetic tree of *M.sumatrana* and *Mangifera* taxa using maximum parsimony analysis derived from: **A**ITS sequences **B***trn*L-F IGS **C** combination ITS+trnL-F IGS sequences. Numbers below branches showed bootstrap values.

The aligned matrix for the combined analysis comprised 1582 characters, of which 89.6% were conserved region and 4.62% parsimony informative. We found one of the most parsimonious trees with a length of 221 steps, CI of 0.91 and RI of 0.80 (Table 3). Additional analysis of genus *M.sumatrana* to its closely related species based on ITS region of nrDNA and trnL-F IGS of chloroplast DNA using MP methods showed that the cladogram was monophyletic. The strict consensus tree is reconstructed by the parsimony method shown in Fig. 4.

Maximum parsimony analysis of the branch leading to *M.sumatrana* with other *Mangifera* species provided a clear resolution. The *M.sumatrana* Miq. is a unique species found in Sumatra and was treated as a synonym of *M.laurina* Bl., based on morphological characters in the latest classification by Kostermans and Bompard (1993) and palynological characters. Based on molecular analysis, using ITS, *trn*L-F IGS sequence and a combination of both, the results can support different species based on morphological and palynological characters (Fig. 4).

The result of BLAST indicated that *Mangiferasumatrana* Miq. ITS sequences (Genbank acc. no. MF990366.1) and *trn*L-F IGS (Genbank acc. no. MF990366.1) have a high similarity to *M.indica* (Table 4). Corresponding to the tree MP, using ITS and *trn*L-F IGS sequence and data from BLAST parameters, *M.sumatrana* Miq. is not a synonym of *M.laurina* Bl (Fig. 4B).

**Table 4.** BLAST analysis of ITS and *trn*l-F IGS sequences of *Mangiferasumatrana* Miq.

Description	Max score	Total score	Query cover (%)	Ident	Accession
*Mangiferaindica* ITS1 (partial), 5.8S rRNA gene, and ITS2 partial), cultivated variety Dasheri.	841	841	100	98.14	AJ890466.1
*Mangiferaindica* cultivar MKR 8 internal transcribed spacer 1, partial sequence; 5.8S ribosomal RNA gene, complete sequence; and internal transcribed spacer 2, partial sequence.	833	833	99	97.93	OL960632.1
*Mangiferaindica* cultivar Tuong BP small subunit ribosomal RNA gene, partial sequence; internal transcribed spacer 1 and 5.8S ribosomal RNA gene, complete sequence; and internal transcribed spacer.	830	830	100	97.72	MN011941.1
*Mangiferaindica* cultivar Gadung internal transcribed spacer 1, partial sequence; 5.8S ribosomal RNA gene and internal transcribed spacer 2, complete sequence; and large subunit ribosomal RNA gene, partial sequence.	830	830	98	98.11	MH037250.1
*Mangiferalaurina* internal transcribed spacer 1, partial sequence; 5.8S ribosomal RNA gene, complete sequence; and internal transcribed spacer 2, partial sequence.	830	830	100	97.72	MF678508.1
*Mangiferaindica trn*L-*trn*F intergenic spacer, partial sequence; chloroplast.	725	725	97	99.25	MF997590.1
*Mangiferaindica* cultivar Arunika *trn*A-Leu (*trn*L) gene, partial sequence; *trn*L-*trn*F intergenic spacer, complete sequence; and *trn*A-Phe (*trn*F) gene, partial sequence; chloroplast.	723	723	96	99.50	JX185679.1
*Mangiferalalijiwa trn*L-*trn*F intergenic spacer, partial sequence; chloroplast.	684	684	91	99.47	MF997587.1
*Mangiferazeylanica trn*L-*trn*F intergenic spacer, partial sequence; chloroplast.	721	721	96	99.50	MF997591.1
*Mangiferafoetida trn*L-*trn*F intergenic spacer, partial sequence; chloroplast.	723	723	96	99.50	MF997585.1

Identification, using DNA barcodes, shows that *M.sumatrana* is related to *M.indica*, *M.zeylanica*, *M.laurina* and *M.lalijiwa*. Based on floral morphological characteristics, these five species of *Mangifera* are grouped with two distinguishing characteristics: panicles glomerulate (*M.indica* and *M.zeylanica*), while *M.laurina*, *M.sumatrana* and *M.lalijiwa* have non-glomerular panicles. However, *M.laurina* was very different from the distinctive features of conical panicles. Meanwhile, the distinguishing feature of *M.lalijiwa* and *M.sumatrana* species is that the crown shape distinguishes between *M.lalijiwa* and *M.sumatrana* species, which are spherical (*M.lalijiwa*) and semi-circular (*M.sumatrana*) crowns. *M.sumatrana* is different. Based on fruit morphological characteristics, *M.sumatrana* has a fruit shape that is very different from other species, namely the fruit is roundish and flattened, a distinguishing feature which is stable and genetic. The differences in *M.sumatrana* shows clearly that *M.sumatrana* is a different species, not a synonym of *M.laurina*. Hence, we propose that *M.sumatrana* is a distinct species amongst the *M.laurina* complex species.

*M.sumatrana* is a narrowly distributed species. It is only found in central Sumatra, with a population of fewer than 100 individuals. Following the Categories and Criteria of the IUCN Red List (IUCN 2012), we categorise *M.sumatrana* as critically endangered according to criteria B and D.

### ﻿Taxonomic key of *M.sumatrana* and its related species

**Table:** 

1	Panicles glomerulate, horizontal axis	**2**
–	Panicles not glomerulate, semi-erect axis	**3**
2	Leaves lanceolate to oblong, fruits green, ovate-oblong	***M.indica***
–	Leaves spathulate to oblanceolate, fruits yellow orange, cordate	***M.zeylanica***
3	Crown shape semi-circular, leaves semi-drooping on branch, panicles terminal, greenish yellow to yellowish-cream, large up 40 cm long	**4**
–	Crown shape spherical, leaves semi-erect on branch panicles pseudo-terminal, light green, large up 20 cm long	***M.lalijiwa***
4	Panicles conical, medium densely, flowers yellow-orange small sepal 1.3–1.8 mm × 0.7–1 mm, fruit roundish thickened, pulp yellow-orange, fibre texture soft	***M.laurina***
–	Panicles pyramidal, low densely, flowers pale yellow, large sepal 3–3.5 × 1.7–2 mm, fruit roundish flattened, pulp pale yellow, fibre texture rough	***M.sumatrana***

## ﻿Conclusion

*M.sumatrana* has a fruit shape that is very different from other species. Namely, the fruit is roundish and flattened, a distinguishing feature which is stable. *M.sumatrana* also has a prolate spheroidal pollen. Based on phylogenetic analysis, *M.sumatrana* is not in the same clade as *M.laurina*. The present study showed that ITS and *trn*L-F IGS DNA barcode markers in combination can be used as taxon-specific markers for *Mangifera*. The findings of this study support the view that *M.sumatrana* can be treated as a distinct species from *M.laurina*.

## Supplementary Material

XML Treatment for Mangifera sumatrana
